# Using Patient Feedback to Improve Treatment Outcomes for Patients with Congenital Dyserythropoietic Anaemia Type I Receiving Interferon Therapy

**DOI:** 10.3390/jcm15020901

**Published:** 2026-01-22

**Authors:** Karl Frey, Sanja Brolih, Caroline Scott, Nicholas Fordham, Sam Burrows, Nyree Cole, Karen Deem, Christopher Jenkins, Melanie Proven, Christian Babbs, Noemi Bernadette Alice Roy

**Affiliations:** 1 Department of Haematology, Oxford University Hospitals NHS Trust, Churchill Hospital, Old Road, Headington, Oxford OX3 7LE, UK; k.frey@nhs.net; 2 MRC Weatherall Institute of Molecular Medicine, University of Oxford, John Radcliffe Hospital, Oxford OX3 9DU, UK; caroline.scott@ndcls.ox.ac.uk (C.S.); christian.babbs@imm.ox.ac.uk (C.B.); 3 Centre for Medicines Discovery, University of Oxford, Oxford OX3 7FZ, UK; sanja.brolih@cmd.ox.ac.uk; 4 Evalina Children’s Hospital, St Thomas Hospital, London SE1 7EH, UK; nicholas.fordham@nhs.net; 5 Department of Haematology, University Hospitals Plymouth NHS Trust, Derriford Road, Plymouth PL6 8DH, UK; 6 Epsom and St Helier University Hospitals NHS Trust, Sutton SM5 1AA, UK; nyree.cole@uhs.nhs.uk; 7 Portsmouth University Hospitals NHS Trust, Portsmouth PO6 3LY, UK; karen.deem@nhs.net; 8 Aneurin Bevan University Health Board, Newport NP11 5GH, UK; chris.jenkins@wales.nhs.uk; 9 Regional Genetic Laboratory, Oxford University Hospitals NHS Trust, Churchill Hospital, Old Road, Headington, Oxford OX3 7LE, UK; melanie.proven@ouh.nhs.uk

**Keywords:** congenital dyserythropoietic anaemia I, patient response, interferon, PROMS, Congenital Anaemia Network

## Abstract

Congenital dyserythropoietic anaemia type-I (CDA-I) is a rare autosomal recessive disease characterised by ineffective erythropoiesis, haemolysis and non-haematological developmental abnormalities. Its treatment is multifactorial, including the management of anaemia, iron overload and prevention of osteoporosis. The only treatment specific to CDA-I is subcutaneous interferon alpha (IFNα) 2A. This study presents the first summary of all published cases of CDA-I patients (*n* = 33) treated with IFNα and categorises their outcome. We also present new unpublished cases (*n* = 7). Overall, we find that IFNα administration causes a statistically significant mean increase in haemoglobin of 30.7 g/L (*p* < 0.001). However, we note that previous studies do not assess the impact of IFNα therapy on providing symptomatic benefit to patients with CDA-I, or the weight of side effects on their quality of life. We collaborate directly with patients through the organisation Congenital Anaemia Network to establish patient preferences regarding IFNα treatment. We propose a classification framework for the use of IFNα in CDA-I that includes patient-reported outcome measures in addition to grading response according to changes in Hb levels. We believe that the use of this framework will aid standardisation in measuring response to therapy, improve clinical practice and assist in future research.

## 1. Introduction

Congenital dyserythropoietic anaemia type-I (CDA-I) (MIM 607465 and #224120) is a rare autosomal recessive disease characterised by macrocytic anaemia, ineffective erythropoiesis, haemolysis and fairly minor non-haematological developmental abnormalities [1]. Erythroblasts of CDA-I patients display pathognomonic morphological abnormalities that can be identified by electron microscopy (EM), referred to as a ‘spongy’ heterochromatin. Additionally, light microscopy reveals enucleation defects, binuclearity and internuclear bridging. Other non-erythroid haematopoietic lineages are unaffected in CDA-I [2].

While these unique microscopic features appear to be preserved across all patients with CDA-I, there is vast heterogeneity in phenotype expression. A 2006 study by Heimpel et al. showed that the age at diagnosis of 21 patients with CDA-I ranged from 0.1 to 47 years old (median 17.3) [3]. This heterogeneity is not only seen between unrelated patients with different mutations, but even in families with identical mutations. For example, al-Fawaz and al-Mashhadani, 1995, describe two siblings, where one presented symptoms in the neonatal period and the other at 2 years of age [4]. CDA-I patients typically present with symptoms of anaemia, which may be accompanied by jaundice, splenomegaly and complications from iron overload, due to increased iron absorption. Non-haematological abnormalities have also been reported, including syndactyly, short stature, flattened vertebral bodies and early osteoporosis. CDA-I is frequently misdiagnosed as more common congenital anaemias, such as congenital haemolytic anaemia or hereditary spherocytosis [3,5].

At the genetic level, 90% of CDA-I patients harbour biallelic mutations in either of the two known causative genes, *CDAN1* and *CDIN1* [6]. In the remaining 10% of cases, no mutations can be identified in either of these genes, suggesting the presence of a third disease locus. Roy and Babbs, 2019, report 51 known causative mutations in *CDAN1* and 5 in *CDIN1* [1]. No genotype-phenotype correlations have been identified thus far. Pathogenic *CDAN1* alleles were first discovered among the Israeli Bedouin tribal group, and CDA-I remains most prevalent in this population. Worldwide, the reported incidence of CDA-I suggests a frequency of 0.2–4.8 cases per million live births [7].

The management of CDA-I is multidisciplinary. The anaemia can be treated by regular blood transfusions, and although splenectomy has been used, it is of little benefit [8]. Iron overload is managed with iron chelation therapy, or, in non-anaemic individuals, with regular venesections. Most patients receive folic acid to reverse deficiencies caused by haemolysis. Additional specialist input is required for management of non-haematological features of CDA-I, including syndactyly and osteoporosis [9].

The only available treatment specific to CDA-I is the administration of subcutaneous interferon alpha (IFNα) 2A. It is known to effectively raise haemoglobin (Hb) levels and reduce iron absorption, thereby decreasing or eliminating the need for blood transfusions and iron chelation therapy. This was first found inadvertently in a patient with CDA-I receiving IFNα treatment for hepatitis C [1]. Scott et al., 2022, confirmed administration of IFNα raises Hb levels in CDA-I patients and further demonstrates that CDA-I patients receiving IFNα treatment show an increase in the number of E burst forming units (BFU-Es), which are the first progenitor cells committed to the erythroid lineage [10]. Electron microscopy studies show that treatment with IFNα partially reverses the ‘spongy’ heterochromatin appearance [6]. IFNα is now widely used for CDA-I, but there is no strict evidence base for this or for its dosing regimens, because no clinical trials have been conducted in this patient group and dosing has followed that currently recommended for hepatitis C treatment, with no investigations as to whether an alternative dosing regimen would be better suited in CDA-I. Additionally, IFNα carries a wide side effect profile, which makes it difficult for some patients to tolerate [11].

A recent report published by the *James Lind Alliance* highlights the Top 10 priorities for Rare Inherited Anaemias as established by a committee of clinicians, researchers, and patients [12]. This includes two points that are highly relevant to this study:

1. Would a national formal network of clinicians with expertise and/or a national MDT (multidisciplinary team meeting) improve care for patients with rare inherited anaemias?
2. Would a register of all rare inherited anaemia patients in the UK (including data and samples) improve care?

While the National Haemoglobinopathy Registry in England, United Kingdom (UK) should have all CDA-I patients registered, the latest published data purports that there are only 11 patients with CDA-I in England, UK [13], a number that is clearly incorrect and at odds with the predicted 300–400 cases expected from genetic carrier rates [6]. We believe that a formal framework for IFNα therapy in CDA-I would improve patient care and facilitate future research in the field. To achieve this, we collaborate with Congenital Anaemia Network (CAN), an organisation that funds research and supports patients with congenital anaemias to gather the input of people with lived experience of the condition.

## 2. Methods

In this study, we incorporate a systematic literature review about treatment responses in all CDA-I patients who have been published to have received IFNα. We also include results from unpublished cases. In total, we collected 40 CDA-I patient reports: 33 already published reports, representing our retrospective case series, and 7 unpublished CDA-I patient reports. We perform a paired t-test statistic for Hb levels pre and post treatment with IFNα. Finally, we collaborate directly with CDA-I patients and carers to propose new ways in which the therapeutic assessment in CDA-I could be standardised to improve patient care and facilitate future research. This includes the creation of a formal classification system to record efficacy and side effects of IFNα therapy using patient-reported outcome measures (PROMs).

Published cases: We searched the PubMed database comprising all recent biomedical literature from MEDLINE, life science journals, and online books published in English before 5 April 2021 using the following or a combination of the following terms: “congenital dyserythropoietic anaemia I”; “CDA-I”; “interferon”; “interferon alpha” and/or “interferon alpha2”.

Inclusion/exclusion criteria: Patients included in this analysis were diagnosed by positive genetic tests for known CDA-I genotypes or displayed ‘spongy’ heterochromatin on electron microscopy. Cases with a CDA-I diagnosis based solely on light microscopy were excluded from this study.

Unpublished cases: We present new cases of patients under speciality care at the Cancer and Haematology Centre, Churchill Hospital, Oxford, or those referred to the South-Central Genomic Laboratory Hub for diagnosis. We obtained written consent from patients or those with parental responsibility over patients for the anonymous publication of this data.

Patient involvement: We collaborate directly with six patients or parents of patients with CDA-I undergoing IFNα therapy through the charity ‘Congenital Anaemia Network’ (CAN). We organised two meetings with the aim of encouraging patient representation in the creation of a formal framework for monitoring and recording outcomes to IFNα therapy in CDA-I.

## 3. Results

In this study, we review 40 patients with CDA-I who have received IFNα therapy and outline their demographics, genetic data, clinical presentation, and response to treatment. The results are outlined in Table 1 [2,3,5,6,13,14,15,16,17,18,19,20,21,22]. A treatment response categorisation criterion is incorporated under the ‘response’ column. This criterion is described in Section 4 and outlined in Table 2.

All patients were treated with variations in IFNα. Dosing regimens varied and did not appear to be protocol-driven. Patients receiving pegylated IFNα typically only required once weekly injections, while non-pegylated IFNα was typically given tri-weekly. We found no trend or correlation between dosage and frequency of IFNα therapy and response, as long as adherence was consistent. In fact, the authors would like to note the vast heterogeneity and at times incomplete patient reports which combined with low patient number demand a cautious interpretation of the data. Nevertheless, from clinical practice we recognise that there is currently poor guidance on how to dose IFNα and in the absence of clinical trial data, this remains empirical and based on doses published for Hepatitis C or myeloproliferative conditions. Pragmatically, many clinicians titrate the dose to optimise response and reduce side effects, but particularly in children, evidence of how to carry this out would be extremely helpful to patients and clinicians alike.

We also found no correlation between the type of mutation and treatment outcomes. Overall, five patients (patients 14, 17, 18, 19, 21) were reported as not having responded to treatment. In the case of patient 14, this can be attributed to low treatment compliance and a self-purchased IFNα. Therefore, this patient was excluded from further data analysis. Patients 17–19 are siblings born to consanguineous parents of Kuwaiti origin, all harbouring the L178Q mutation in *CDIN1*. Interestingly, patient 16 displays the same mutation but showed a significant increase in Hb upon IFNα, suggesting genetic modifiers may play a role. Patient 21 underwent genetic testing for CDA-I, but no mutations were found in *CDAN1* or *CDIN1*. A diagnosis was made based on electron microscopy. Given that the detection of ‘spongy’ heterochromatin is diagnostic of CDA-I, this finding supports the hypothesis that there is a third disease locus, as suggested by Olijnik et al. 2021 [6]. Intriguingly, this patient did not respond to IFNα, potentially linking poor treatment outcomes to mutations in a yet undiscovered CDA-I disease gene.

**Figure 1:**
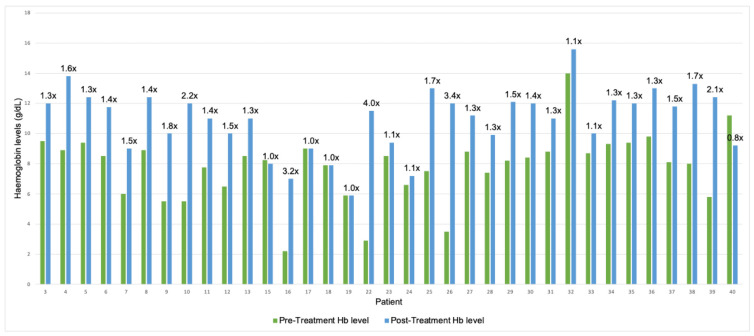
Haemoglobin Responses to IFNα Treatment. For each patient, the green bar indicates pre-treatment Hb levels. Blue bars represent post-treatment Hb levels. Fold change in Hb level pre/post treatment is indicated above the bars. Hb: Haemoglobin; x: fold.

Our extensive literature search highlighted that most studies reported outcomes primarily based on pre- and post-treatment Hb levels. Figure 1 highlights the pre- and post-treatment Hb levels where available. In patients where these were documented, we found a mean increase of 30.7 g/L following IFNα therapy, or on average a 1.5-fold increase (mean pre-treatment: 79.0 g/L; mean post-treatment 109.7 g/L; *n* = 36). We performed a paired t-test to prove statistical significance in this effect (*p* < 0.001). No other significant conclusions could be drawn from these comparisons, although we note that IFNα treatment at an early age seems to have a more pronounced effect on Hb levels. From our clinical experience, response to IFNα is usually rapid (within 4–8 weeks), and lack of response after this time period usually predicts complete unresponsiveness, and the therapy should probably be stopped at that stage. While most clinicians, especially when starting IFNα in young children who have been transfusion dependent, monitor bloods weekly, there is probably no need to do this, and a monthly blood count should be sufficient. Liver function tests should also be carried out, and a rise in ALT can be seen, which is usually transient but may require dose reduction in IFNα.

Crucially, no study considered patient preferences or a clinically validated PROMs reporting system as useful parameters to define treatment success. We also found that side effect reporting was inconsistent and would benefit from standardisation. We therefore collaborated directly through CAN with six patients (or carers) with CDA-I receiving IFNα therapy. We organised an online discussion forum to collect PROMs and later re-evaluated these at the yearly national CAN CDA-1 patient conference 2025.

Using an online questionnaire, overall experience with IFNα was quantified on a 1–5 Likert scale, with a mean of 3.3/5 (*n* = 6). Positive effects of IFNα included increased energy levels, reduced need for iron chelation and blood transfusions, and a reduction in vomiting and pain flares. All six patients reported experiencing side effects, namely fever, flu-like symptoms, headaches, muscle pains, diarrhoea, and bruising at the injection site. We asked patients to rank the following four questions relating to their treatment based on patient priority. These were selected according to our previous online discussion with the same patient group. They are outlined below in preferential order:

1. Are my symptoms (e.g., fatigue) improving with interferon?
2. Can I stop other treatments (e.g., iron chelation, blood transfusions) because of interferon?
3. Will interferon reduce my risk of CDA-I-related problems in the future?
4. Are my Hb levels better after interferon?

It is noteworthy that the impact of the treatment on Hb levels, the only consistently recorded treatment outcome in publications, is of least importance to patients, suggesting that PROMs should be recorded as standard in future assessments and treatment responses.

## 4. Discussion

Our survey unanimously highlights that symptomatic improvement is the most important aspect of IFNα therapy for our patients. They described frustration over the heavy emphasis on measuring Hb levels throughout their treatment journey, when this frequently did not correspond to improvements in fatigue. We set out to devise treatment response criteria, partly based on objective quantifiable data highlighted by our literature search and partly based on a subjective assessment of symptoms and side effects, as deemed important to our patient group. This is outlined in Table 2. Table 1 incorporates these scoring criteria under the ‘response’ column.

1. Quantifiable criteria

Hb levels provide a quantitative measure of the effect of IFNα. Symptoms of CDA-I can be largely attributed to anaemia, but Hb alone is not sufficient to predict symptoms. Fatigue may result from ineffective haematopoiesis and/or haemolysis in addition to Hb levels. Other blood parameters of interest include iron studies to monitor iron overload. Changes in MCV are unlikely to hold clinical value. A reduction in the requirement for blood transfusions and iron chelation can also be quantified. This was of second highest importance to patients in our survey. Blood transfusions are time-consuming and require frequent intravenous access, and some patients report that IFNα use has given them time back in their daily routine. Additionally, iron chelation therapy has a wide side effect profile, including visual changes, rashes, gastrointestinal upset, dizziness, and abdominal cramping.

2. PROMs

Ideally, a clinically validated score should be utilised to quantify fatigue before and after treatment. The FACIT-Fatigue (Functional Assessment of Chronic Illness Therapy-Fatigue) scale assesses self-reported fatigue and its impact on daily activities and function [26]. It was developed for a more precise evaluation of fatigue associated with anaemia in cancer patients, but a recent randomised controlled trial has supported the use of this score in iron deficiency anaemia with good reliability [27]. Further research is required to clinically validate a fatigue score for use in congenital anaemias.

3. Side Effects

IFNα is well known to cause a range of side effects, most commonly fever, flu-like symptoms, abdominal pain, and diarrhoea. Only 10/39 patients in this study were reported to tolerate IFNα well, with no dose correlation evident from available data. Our collaboration with the patient group has highlighted that these common IFNα side effects are experienced regularly, with heterogeneity as to whether these attenuate over time. In addition, patients reported they took more precautions ahead of their injections, such as pain relief medication or booked time off work, or in some cases, lowered their dosage to prioritise adherence. In fact, from patient feedback, these side effects pose a real-life obstacle to adherence and should therefore carry significant weight in the response evaluation. Patients also noted that information about side effects in the literature and on patient information leaflets commonly focuses on immediate to short term side effects, while patients receive little information on long term effects of IFNα use. This is likely because IFNα is most commonly used for the treatment of Hepatitis B and C as a 6 to 12-month course. Ideally, both short- and long-term side effects of IFNα therapy would be assessed using a personalised IFNα-therapy side-effects scale. In the absence of such a validated clinical scale, especially for Congenital Anaemias, we suggest that adverse event reporting tools such as the Common Terminology Criteria for Adverse Events (CTCAE) could be used for the categorisation criteria [28].

The use of this categorisation criteria would allow clinicians and researchers to collect data more effectively and better understand how patients respond to IFNα and the reasons behind discontinuation of treatment. It could also be used to evaluate any novel therapies that are studied in CDA-I in the future.

In the absence of PROMs from already published reports, we have used the quantifiable criteria to grade the treatment responses of patients in our study. According to these, 32/39 patients in our data analysis (82%) exhibited an overall positive response to IFNα therapy.

CDA-I remains a rare condition, and the scarcity and heterogeneity of the data demand caution when interpreting results. Still, we believe that the use of our proposed system, which includes PROMS, will promote good practise by providing clinicians with a standardised framework. This will allow qualitative and quantitative evaluation of treatment response, facilitating future research in this field. Moreover, it provides the patient with tangible information about their therapeutic response and empowers them to make autonomous decisions about their care. The clinical validation of a fatigue scale, such as FACIT-fatigue for congenital anaemias, would facilitate PROMs for IFNα therapy in patients with CDA-I. We will collaborate with CAN to achieve this goal.

## Figures and Tables

**Table 1:** CDA-I Patients with Identified Mutations—IFNα Response.

Patient	Dem	Genomic Region of Mut	Mutation	Hom/Het	Pre-Treatment	Treatment Response	Response	Treatment Regime	Side Effects	Other Clinical Features	Reference
1	20 F	*CDAN1* Exon 2 *CDAN1* Exon 14	F52L P672L	Com. Het	Transfusion dependent for 19 years	Hb: 101–124 g/L MCV: 91–93 fL Transfusion independent	B1	IFNα 2a 3 × 3 MU/wk	NS	Pulmonary hypertension, digital clubbing, osteoarthritis	(Niss et al. 2021) [2]
2	1 M	*CDAN1* Exon 2 *CDAN1* Exon 23	F52L E1009GfsTer10	Com. Het	Transfusion dependent for 12 months	Hb: 78–101 g/L MCV: 89–91 fL Transfusion-independent for 4 years since discontinuing IFN treatment	B1	IFNα 2a 3 × 3 MU/wk Discontinued after 2 yrs	NS	Hepatomegaly, pulmonary hypertension	(Niss et al. 2021) [2]
3	48 F	*CDAN1* Exon 12 *CDAN1* Exon 25	N599S A1086PfsTer11	Com. Het	Hb: 90–100 g/L MCV: 100–120 fL Treated with iron chelation	Hb: 120 g/L Reduced need for chelation	A1 C2	IFNα 2a 3 × 3 MU/wk Reduced to 2 MU/wk	Symptomatic neutropenia	Splenomegaly, syndactyly, liver siderosis, and cirrhosis	(Heimpel et al. 2006) [3]
4	F	*CDAN1* Exon 14 *CDAN1* Exon 20	R682X L915-L922 del	Com. Het	Hb: 89 g/L MCV: 103 fL	Hb: 138 g/L MCV: 104 fL	A1	IFNα 2	NS	NS	(Olijnik et al. 2021) [6]
5	51 F	*CDAN1* Exon 14 *CDAN1* Exon 23	R725W 3133 TT insertion	Com. Het	Hb: 94 g/L MCV: 106 fL	Hb: 119–129 g/L	A1	IFNα 2b 3 × 3 MU/wk	Reduced quality of life	NS	(Heimpel et al. 2006) [3]
6	42 F	*CDAN1* Exon 25 *CDAN1* Exon 26	A1086P fsTer11 P1130L	Com. Het	Hb: 80–90 g/L MCV: 110–120 fL Treated with iron chelation	Hb: 105–130 g/L Reduced need for chelation	A2 C2	IFNα 2b 3 × 3 MU/wk	Asymptomatic neutropenia	Syndactyly	(Heimpel et al. 2006) [3]
7	6 wksF	*CDAN1* intron 12	IVS −12 + 5G > A	Het	Hb: 60 g/L Transfusion dependent	Hb: 80–100 g/L Transfusion independent	A2 B1	IFNα	NS	Nail hypoplasia, acral dysostosis	(Shalev et al. 2004) [5]
8	54 F	*CDAN1* Exon 28	(−10 + 31bp) del	Het	Hb: 89 g/L MCV: 106 fL	Hb: 114–134 g/L	A1	IFNα 2b 3 × 3 MU/wk	Reduced quality of life	NS	(Heimpel et al. 2006) [3]
9	16 F	*CDAN1* Exon 24	R1042W	Hom	Hb: 50–60 g/L MCV: 94 fL	Hb: 100 g/L MCV: 96 fL	A2	Pegylated IFNα 2a 180 µg/wk	Flu-like symptoms, myalgia	Thalassaemia minor trait	(Abu-Quider et al. 2020) [14]
10	16 M	*CDAN1* Exon 24	R1042W	Hom	Hb: 50–60 g/L MCV: 91 fL	Hb: 120 g/L, MCV: 84 fL	A2	Pegylated IFNα 2a 180 µg/wk	Flu-like symptoms, myalgia	Delayed puberty, growth hormone deficiency	(Abu-Quider et al. 2020) [14]
11	4 F	*CDAN1* Exon 24	R1042W	Hom	Hb: 70–85 g/L MCV: 86 fL	Hb: 110 g/L, MCV: 84 fL	A2	Pegylated IFNα 2a 90 µg/wk	Flu-like symptoms, myalgia	NS	(Abu-Quider et al. 2020) [14]
12	5 F	*CDAN1* Exon 24	R1042W	Hom	Hb: 65 g/L MCV: 89 fL	Hb: 100 g/L MCV: 88 fL	A2	Pegylated IFNα 2a 90 µg/wk	Flu-like symptoms, myalgia	NS	(Abu-Quider et al. 2020) [14]
13	11 F	*CDAN1* Exon 24	R1042W	Hom	Hb: 75–95 g/L MCV: 88 fL	Hb: 100–120 g/L. MCV: 86 fL Returned to original baseline after discontinuation of IFN	A2	Pegylated IFNα 2a 90 µg/wk	Flu-like symptoms, moon-face, abdominal distention	NS	(Abu-Quider et al. 2020) [14]
14	12 F	*CDAN1* Exon 24	R1042W	Hom	Hb: 65–90 g/L MCV: 86 fL	No Hb response, MCV: 87 fL *	A0	Pegylated IFNα 2a 180 µg/wk	Flu-like symptoms, myalgia	Bone manifestations	(Abu-Quider et al. 2020) [14]
15	5 F	*CDAN1* Exon 24	R1042W	Hom	Hb: 55–110 g/L MCV: 89 fL	Hb: 80 g/L MCV: 97 fL	A0	Pegylated IFNα 2a 90 µg/wk	Flu-like symptoms, myalgia	NS	(Abu-Quider et al. 2020) [14]
16	4 M	*CDIN1* Exon 8	L178Q	Hom	Hb: 22 g/L at birth	Hb: 70 g/L	A2	IFNα 2a 67.5 µg/wk reduced to 45 µg/wk	Neutropenia	Syndactyly, ventricular septal defect, growth delays, learning disability, jaundice	(Rathe et al. 2018) [15]
17	29 F	*CDIN1* Exon 8	L178Q	Hom	Hb: 90 g/L MCV: 98 fL Transfusion dependent	No Response	A0 B0	IFNα	NS	Jaundice, syndactyly, hepatosplenomegaly, growth delays, hand and foot hypoplasia	(Babbs et al. 2013) [23]
18	20 M	*CDIN1* Exon 8	L178Q	Hom	Hb: 79 g/L MCV: 84 fL Transfusion dependent	No Response	A0 B0	IFNα	NS	Jaundice, congenital ptosis, hepatomegaly, growth delays, hand and foot hypoplasia	(Babbs et al. 2013) [23]
19	17 M	*CDIN1* Exon 8	L178Q	Hom	Hb: 59 g/L MCV: 79 fL Transfusion dependent	No Response	A0	IFNα	NS	Jaundice, hepatosplenomegaly, growth delays, hand and foot hypoplasia	(Babbs et al. 2013) [23]
20 †	45 F	*CDIN1* Exon 5	Y94C	Hom	Transfusion dependent	Reduced need for transfusions	B2	IFNα	Thrombocytopenia	NS	(Babbs et al. 2013) [23] (Scott et al. 2020) [16]
21	15 M	CDA-1 confirmed by EM (no *CDAN1* or *CDIN1* mutations)	n/a		Hb: 90 g/L MCV: 87 fL	No response	A0	IFNα 2	NS	Splenomegaly	(Olijnik et al. 2021) [6]
22	32 F	NS	n/a		Hb: 29 g/L MCV: 91 fL Transfusion dependent Treated with iron chelation	Hb: 115 g/L Transfusion independent	A2 B1	IFNα 3 × 3 MU/wk	Well tolerated	Jaundice, splenomegaly	(Roda et al., 2002) [17]
23	15 F	NS	n/a		Hb: 85 g/L MCV < 80 fL	Hb: 94 g/L	A3	IFNα 2b 4.5 MU/wk	Flu-like symptoms	Beta thalassaemia trait	(Agrigento et al. 2017) [18]
24	50 F	NS	n/a		Hb: 66 g/L MCV < 80 fL Transfusion dependent (20 days)	Hb: 72 g/L Transfusion dependent (49 days)	A3 B2	IFNα 2b 3 × 3 MU/wk	Well tolerated	NS	(Agrigento et al. 2017) [18]
25	28 F	NS	n/a		Hb: 70–80 g/L Transfusion dependent (monthly)	Hb: 130 g/L Normalised EM Transfusion independent	A1 B1	IFNα 2a 3 × 3 MU/wk Then pegylated IFNα 2b 2 × 2 MU/wk	Well tolerated	NS	(Lavabre-Bertrand et al. 1995) [19]
26	14 mosF	NS	n/a		Neonatal Hb: 35 g/L Transfusion dependent (monthly) EM: 62% shc	Hb: 120 g/L EM (18 mos): 30% shc EM (23 mos): 24% shc Transfusion independent	A1 B1	IFNα 3 × 1 MU/wk, then 2 MU/wk	Well tolerated	NS	(Parez et al. 2000) [20]
27	14 F	NS	n/a		Hb: 88 g/L MCV: 100 fL Transfusion dependent	Hb: 112 g/L MCV: 92 fL Transfusion independent	A2 B1	IFNα 2a 3 × 3 MU/wk reduced to 2 × 3 MU/wk	Well tolerated	Syndactyly, failure to thrive, splenomegaly, gallstones	(Roda et al. 2002) [17]
28	3 mosM	NS	n/a		Hb: 74 g/L Transfusion dependent	Hb: 99 g/L Transfusion independent EM: reduced % shc	A2 B1	IFNα 3 × 1 MU/wk	Well tolerated	Consanguine parents	(Yarali et al. 2005) [21]
29	30 F	NS	n/a		Hb: 82 g/L EM: 58% shc Treated with iron chelation	Hb: 121 g/dL EM: 16% shc Splenomegaly reversed (8cm to 5cm)	A1	IFNα 2a 3 × 3 MU/wk	Well tolerated	Splenomegaly	(Wickramasinghe 1997) [22]
30	31 M	NS	n/a		Hb: 84 g/L MCV: 77 fL	Hb: 120 g/L Splenomegaly reversed (12cm to 3cm)	A2	IFNα 3 × 3 MU/wk	Well tolerated	Splenomegaly	(Shamseddine 2000) [24]
31	64 F	NS	n/a		Hb: 88 g/L MCV: 85 fL	Hb: 110 g/L	A2	IFNα 3 × 3 MU/wk	Nausea, Myalgia	Splenectomy at age 22	(Shamseddine 2000) [24]
32	34 F	*CDAN1* Exon14 *CDAN1* Exon 20	P672L E894VfsTer109	Com. Het	Hb: 140 g/L Ferritin 2928, 4-weekly venesection for iron overload	Hb: 113–200 g/L reduced venesection requirement	A1 C2	Pegylated IFNα 2a 135 µg/wk	Rash, joint pain, diarrhoea, polycythemia	Splenectomy, cholecystectomy, iron overload, IBS, abdominal pain, vitamin D deficiency, low BMI, skeletal abnormalities	(Olijnik et al. 2021) [6]
33	11 M	*CDAN1* Exon 6 *CDAN1* Exon 15	D365N Q754R	Com. Het	Hb: 87 g/L MCV: 75.2 fL Ferritin 2145.8 Transfusion dependent	Hb stable at 100 g/L, Transfusion independent	A2 B1	Pegasys IFNα 105 mcg/wk	?Growth retardation	Short stature, global developmental delay, beta thalassaemia trait, hypospadias, undescended testes, choanal atresia, abdominal pain	(Olijnik et al. 2021) [6]
34	28 F	*CDAN1* Exon 13	R623W	Hom	Hb: 93 g/L MCV: 93 fL	Hb: 122 g/L	A1	IFNα 2a 180mcg weekly	Severe muscle pain, unable to get out of bed	Thalassaemia minor trait, asthma, congenital hypothyroidism, joint pain	
35	F	*CDAN1* Exon 14 *CDAN1* Intron 22	P672L Ivs22 + 5G to C	Com. Het	Hb: 94 g/L MCV: 96 f	Hb: 120 g/L	A1	IFNα	NS	On/off treatment	
36	32 F	*CDAN1* Exon 14 *CDAN1* Exon 2	P672L F52L	Com. Het	Hb: 98 g/L. MCV: 104.2 fL Ferritin 501.4 Transfusion independent	Hb 130 g/L, MCV 85.1, Transfusion independent Good symptomatic response	A1 B1	Pegylated IFNα 2a 65 µg/wk	Well tolerated	Neonatal Jaundice, syndactyly, leg length discrepancy, osteoporosis	
37	4 M	*CDAN1* Exon 14 *CDAN1* Exon 2	R725W P51L	Com. Het	Hb: 70–92 g/L Transfusion dependent (4 weekly) Ferritin 928	Hb: 118 g/L Transfusion independent Ferritin 1223	A2 B1	Pegylated IFNα 2a 90 µg/wk	Well tolerated	Hydrops fetalis in 2nd trimester, 4 intra-uterine transfusions	
38	41 F	*CDAN1* Exon 13 *CDAN1* Exon 13	P632L A644S	Com. Het	Hb: 80 g/L Transfusion requirement only during pregnancy. Post-splenectomy: Hb: 102 g/L MCV: 110 fL	Hb: 133 g/L	A1	Pegylated IFNα 2a 180 µg/wk	Deranged liver function tests—LFT 25 to 120. Led to dose reduction. Asymptomatic	Transfusion requirement at birth, developmental delay in childhood, congenital heart disease	
39	14 mosM	*CDAN1* Exon 12 *CDAN1* Exon 14	N599S P694L	Com. Het	Hb 58 g/L at birth, requiring occasional transfusions	Hb: 124 g/L, transfusion independent for 2 months	A2	IFNα	Well Tolerated	Pulmonary HTN	
40	29 F	*CDAN1*	Q904 * c.1466T > C (M489T) or c.2833G > C (V945L)	Com. Het	Hb: 112g/L MCV: 104 fL Ferritin 445	Hb: 92 g/L MCV 109.5 fL ferritin 555	A2	Pegylated IFNα 2a 180 µg/wk	Well Tolerated—great symptomatic response, improvement in nausea, abdominal pain, and diarrhoea. Best patient has felt in 5 years.	Abdominal Pain, Recurrent Syncope, Asthma, Gall Stones, Gilbert Syndrome, Postnatal Depression, Has Haemochromatosis gene heterozygous mutation.	

Age is recorded at the time of IFNα treatment initiation and is in years unless otherwise specified; yrs: years; wks: weeks; mos: months; M: male; F: female; Hom: Homozygous; Het: Heterozygous; Com. Het: Compound Heterozygous; Mut: Mutation; Dem: Demographic; NS: Not specified; † Patient death due to COVID-19; * Patient self-purchased IFN; n/a: not applicable. Treatment response refers to what is reported in the paper cited. “Response” refers to our grading of the response based on our proposed classification outlined in Table 2.

**Table 2:** Proposed Categorisation Criteria for IFNα Response in CDA-I patients.

Criteria	
Haemoglobin Response (HbA)	Requires durable response > 6 months
A_1_	Hb increase to non-anaemic levels 120–160 g/L (females) or 130–170 g/L (males)
A_2_	Hb increase > 20 g/L
A_3_	Hb increase < 20 g/L
A_0_	No significant response
Transfusion Requirement (Blood)	Requires durable response > 6 months
B_1_	Transfusion independence
B_2_	Reduced transfusion requirement/frequency
B_0_	No significant response
Iron Chelation Status (Chelation)	Requires durable response > 6 months
C_1_	Independent of iron chelation AND venesection
C_2_	Reduced chelation requirements OR reduced venesection requirements
C_0_	No significant response
Patient Reported Outcomes	Requires durable response > 6 months
Meaningful symptomatic response [25]	FACIT-Fatigue scale improvement of ≥ 3
Side effects	Graded as per the Common Terminology Criteria for Adverse Events (CTCAE)
Grades 1–5	Or a specific patient-feedback-led IFN therapy scale

No significant response is defined by a lack of response or any response that doesn’t meet the above criteria.

## Data Availability

Any further information about the presented data in this study is available upon request.
